# Pomegranate-derived phosphorus, nitrogen-doped carbon nanoflower/nanofiber hybrids and quasi-spherical dot stabilized Pickering emulsions with low-DS amphiphilic CMC for dual-mode detection of pathogenic bacteria and their metabolic pH signatures

**DOI:** 10.1039/d6ra02135b

**Published:** 2026-07-29

**Authors:** Hebat-Allah S. Tohamy

**Affiliations:** a Cellulose and Paper Department, National Research Centre 33 El Bohouth Str., P. O. 12622, Dokki Giza Egypt hebasarhan89@yahoo.com

## Abstract

This study introduces a sustainable dual-waste valorization strategy, transforming agricultural residues (*e.g.* pomegranate skin and sugarcane bagasse) into a multifunctional smart diagnostic platform for the real-time detection of pathogenic bacteria. Utilizing a microwave-assisted hydrothermal route, we engineered a morphological evolution from quasi-spherical nitrogen-doped carbon dots (NCQDs) to hierarchical phosphorus and nitrogen co-doped carbon nanoflower/fiber hybrids (PNCNFs). The strategic introduction of phosphorus induced lattice strain and electronic reconfiguration, resulting in a significant increase in the hydrophilic–lipophilic balance (HLB) from 5.57 to 8.95. This elevated HLB, coupled with a tailored low-degree of substitution (DS 0.4) carboxymethyl cellulose (CMC) from bagasse, optimized the stability of Pickering nanoemulsions at the rosemary essential oil/water interface. The PNCNF hybrids demonstrated a superior surface-to-volume ratio, facilitating high-density functional site accessibility for dual-mode sensing. This platform provided synchronized colorimetric and fluorometric responses to the metabolic pH signatures of *Escherichia coli* and *Staphylococcus aureus*. DFT calculations and fluorescence dynamics confirmed that the P,N-doped framework possesses high kinetic reactivity and a pH-switchable electronic structure. Beyond detection, the system showed enhanced antibacterial efficacy through the synergistic action of the hierarchical petals and encapsulated bio-actives. This research demonstrates that the controlled manipulation of biomass-derived molecular precursors provides a viable pathway for developing multifunctional nanophotonic sensors, with potential applications for real-time monitoring in food safety and clinical diagnostics.

## Abstract 2

Sustainable upcycling of pomegranate peel and bagasse into P,N-doped carbon hybrids. Pickering nanoemulsions provide colorimetric and fluorometric detection of bacterial pH signatures.

## Graphical abstract

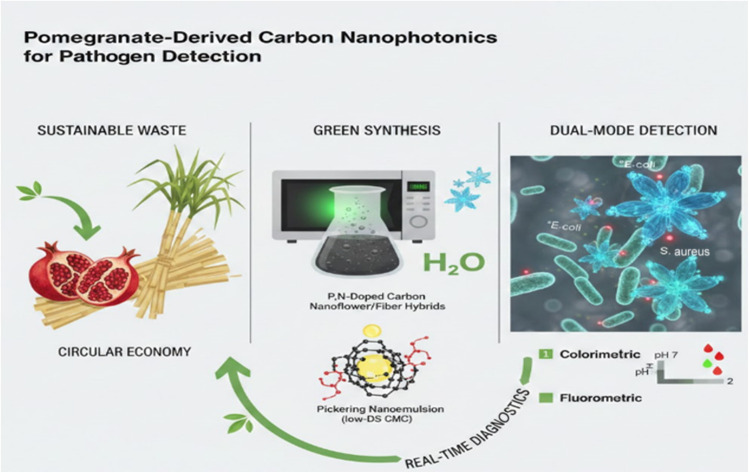

## 1. Introduction

Historically, the synthesis of carbon-based nanomaterials particularly bulk carbons and graphene-related structures was dominated by top-down methods such as arc discharge, laser ablation, or chemical oxidation, which often require extreme energy inputs and the use of harsh mineral acids for surface passivation.^1–5^ However, the landscape of nanocarbon research has evolved significantly with the rapid emergence of bottom-up approaches. For the specific class of carbon quantum dots (CQDs), techniques such as hydrothermal, solvothermal, microwave-assisted, and pyrolytic carbonization have become widely adopted, offering precise control over surface chemistry and morphology.^6–9^ This study introduces a dual-waste valorization strategy by integrating sugarcane bagasse (SCB) and pomegranate (*Punica granatum*) skin waste into a multifunctional diagnostic platform. Pomegranate biomass is rich in punicalagins and ellagitannins complex polyphenols that serve as an effective molecular scaffold during microwave-assisted hydrothermal carbonization.^10,11^ The selection of pomegranate (*Punica granatum*) as a sustainable biomass precursor is based on its rich biochemical composition. Unlike simpler sugar-based precursors, pomegranate biomass contains high concentrations of punicalagins and ellagitannins complex, multi-ringed polyphenols which serve as an effective molecular scaffold during the microwave-assisted hydrothermal process.^12–14^ The phenolic hydroxyl and galloyl groups act as natural capping agents, regulating nucleation and preventing uncontrolled aggregation.^15,16^ Furthermore, the strategic co-doping of nitrogen (N) and phosphorus (P) induces localized lattice strain due to the larger atomic radius of phosphorus. This electronic reconfiguration facilitates a morphological transition from zero-dimensional (0D) quasi-spherical dots to one-dimensional (1D) nanofibers and three-dimensional (3D) hierarchical nanoflowers, significantly increasing the surface-to-volume ratio and the density of accessible active sites.^17,18^ To stabilize these architectures within an aqueous medium, we utilize carboxymethyl cellulose (CMC) derived from SCB. With a controlled degree of substitution (DS 0.4), the CMC exhibits an amphiphilic character that allows it to function as a soft-particle co-stabilizer at the oil–water of the Pickering nanoemulsions. This creates a robust network that preserves the structural integrity of the P,N-doped hybrids while preventing coalescence.

In the architecture of carbon nanomaterials, the ability to transition from zero-dimensional (0D) structures to higher-dimensional geometries represents a significant leap in functional design.^19^ While the vast majority of carbon quantum dots (CQDs) reported in literature are restricted to simple, quasi-spherical shapes, this research highlights a sophisticated evolution in morphology control. The fundamental logic behind this structural shift lies in the synergistic effect of dual-element doping. By strategically introducing P atoms alongside N atoms, the traditional isotropic growth of the carbon core is disrupted. The larger atomic radius of phosphorus creates lattice strain and alters the electronic density of the carbon matrix, acting as a chemical director that forces the material to bloom into 1D nanofibers and 3D hierarchical nanoflowers.^20–23^ This transition from dots to flowers is not merely aesthetic; it is a critical optimization for high-performance sensing. These complex, branched architectures offer a vastly superior surface-to-volume ratio compared to their spherical counterparts. In the context of bacterial detection, the petals of the nanoflowers and the elongated bodies of the nanofibers provide a much larger physical platform for cellular attachment and interaction. Furthermore, the jagged, hierarchical edges create a high density of accessible active sites and functional groups. For dual-mode pH sensing, this means more points of contact for protonation and deprotonation, leading to faster response times and significantly enhanced sensitivity. By moving away from simple spheres, we create a more interactively aggressive material capable of detecting subtle environmental changes and microbial presence with much greater precision.

The reliability of analytical sensors is often compromised by environmental noise, such as background autofluorescence or fluctuations in excitation intensity, which can lead to false readings in complex biological matrices.^24–28^ To address this, we developed a dual-mode sensing platform that integrates colorimetric (naked-eye) and fluorometric (light emission) signaling.^29,30^ The global shift toward green synthesis has redefined the boundaries of nanotechnology, transforming agricultural byproducts into functional materials. This study introduces a dual-waste valorization strategy by integrating sugarcane bagasse (SCB) and pomegranate (*Punica granatum*) skin waste into a cohesive diagnostic platform. While pomegranate waste provides a rich source of polyphenolic compounds for synthesizing P,N-doped carbon nanoflower/fiber hybrids *via* microwave-assisted hydrothermal carbonization, the SCB provides the structural framework for a specialized low-degree of substitution (DS 0.4) carboxymethyl cellulose (CMC). Unlike conventional soluble polymers, this tailored CMC acts as a soft-particle co-stabilizer, engineered to reside at the Pickering nanoemulsions. The intersection of microbiology and material science presents a unique challenge: the need for real-time, autonomous monitoring of pathogenic threats. A critical biological link in this endeavor is the fact that bacteria inherently alter the chemical profile of their microenvironment through metabolic processes.^9,25^ As microbial colonies proliferate, they consume nutrients and release acidic or alkaline metabolic by-products, causing localized fluctuations in the surrounding pH levels.^31,32^ By engineering a material that is sensitive to these specific chemical signatures, we can bridge the gap between sensing and tangible biological activity. The P,N-doped carbon nanoflowers and fibers act as an integrated bridge, where the structural petals and high-surface-area fibers serve as docking sites for bacterial cells while simultaneously transducing the resulting pH shifts into measurable optical signals. The ultimate goal of this research is to move beyond passive observation and toward the creation of a smart diagnostic platform with broad implications for food safety and clinical diagnostics. In food industry applications, such a tool could serve as an intelligent packaging indicator, alerting consumers to spoilage through a visible color change or fluorescence shift triggered by bacterial outgrowths. In a clinical setting, this dual-mode capability allows for the rapid, highly sensitive detection of infection markers even at low concentrations. By synthesizing a single material that detects both the bacteria themselves and the metabolic pH changes they induce, we are establishing a sophisticated, multifunctional sensing paradigm. This smart nanosystem not only accelerates the detection timeline but also provides a more comprehensive understanding of microbial behavior, ensuring higher standards of public health and safety.

## 2. Materials and methods

### 2.1. Materials

Pomegranate skin wastes were sourced from the Egyptian market. Di-sodium hydrogen orthophosphate anhydrous was purchased from SD Fine-Chem. The monochloro-acetic acid, sodium hydroxide, urea, and calcium chloride were purchased from Sigma-Aldrich (St. Louis, MO, USA). All materials were used as received without further purification. All other analytical grade reagents were used as received. Sugarcane bagasse (SCB) was kindly provided by Quena Company for Paper Industry, Egypt.

### 2.2. Synthesis of pomegranate skin wastes-derived carbon nanostructures

The carbon nanostructures were synthesized using a multi-mode domestic microwave system (900 W output). To ensure reproducibility, the reaction volume was maintained at a constant 100 mL in a 250 mL borosilicate glass vessel. While the system operates without integrated temperature and pressure sensors, consistency was achieved by standardizing the irradiation time, power output, and the concentration of chemical activators (NaOH), which facilitate rapid dielectric heating and localized structural reorganization. The selection of 900 W microwave power was dictated by the structural complexity of the pomegranate skin biomass. As a dense, lignocellulosic-rich residue, the pomegranate matrix requires high-intensity dielectric heating to overcome its inherent recalcitrance and trigger the rapid dehydration and carbonization necessary for forming carbon nanostructures. Preliminary trials indicated that maximum power (900 W) was required to ensure complete hydrothermal conversion within the 100 mL aqueous reaction volume, thereby preventing incomplete carbonization and ensuring the morphological uniformity of the final products.

#### 2.2.1. Synthesis of quasi-spherical nitrogen-doped carbon dots (NCQDs)

Fresh red pomegranate skin wastes were finely chopped and homogenized. A dispersion was prepared by adding 4 g of the pomegranate skin wastes biomass into 100 mL of deionized water. To this, 4 g of NaOH and 4 g of urea were added. The NaOH acted as a chemical activator to facilitate the breakdown of the pomegranate skin wastes' cellulosic and anthocyanin structures, while urea provided an additional N source to ensure high-density surface doping. The mixture was subjected to microwave irradiation at 900 W for 10 minutes.^33^ During this phase, the organic molecules underwent rapid dehydration and carbonization, resulting in the formation of ultra-small, quasi-spherical particles. Following synthesis, the mixture was cooled to room temperature, passed through filter paper to remove large-scale carbonaceous aggregates and to isolate the NCQDs from unreacted precursors. The final product was washed three times with deionized water and ethanol to ensure purity.

#### 2.2.2. Synthesis of P,N-doped carbon nanoflower/nanofiber hybrids (PNCNF)

Similar to the NCQDs, 4 g of red pomegranate skin wastes biomass was dispersed in 100 mL of deionized water. In addition to 4 g of NaOH, 4 g di-sodium hydrogen orthophosphate anhydrous and 4 g of urea. The introduction of P disrupts the uniform growth seen in spherical dots. The lattice strain induced by the larger P atoms, combined with the natural fibrous template of the pomegranate skin wastes, facilitates the hierarchical growth of nanofibers and the assembly of nanoflowers. The solution was treated at 900 W for 10 minutes. Post-synthesis, the crude product was subjected to the same standardized purification protocol: filtration through a high-retention filter paper to remove macro-aggregates. The isolated hierarchical hybrids were washed repeatedly with deionized water and ethanol to remove residual phosphate and urea, and subsequently vacuum-dried at room temperature for 12 hours.

### 2.3. Dual-mode pH-responsive sensing and colorimetric evaluation

To evaluate the fundamental pH-responsiveness and electronic switching capabilities of the synthesized NCQDs and PNCNFs, fluorescence measurements were recorded across a broad range (pH 2.0–12.0). The optical responsiveness of the NCQDs and PNCNFs to chemical stimuli was evaluated by monitoring their behavior across a broad pH spectrum, representing acidic, neutral, and alkaline conditions (pH 2.0, 7.0, and 12.0, respectively). The pH of the aqueous dispersions was precisely adjusted using 0.1 M HCl and 0.1 M NaOH to simulate the extreme chemical environments associated with bacterial metabolism and environmental degradation. To ensure thermodynamic equilibrium, each sample was equilibrated for 24 hours prior to measurement. The dual-mode sensing capability was validated through synchronized colorimetric monitoring and fluorometric sensitivity analysis:

• Colorimetric monitoring (naked-eye detection): the transition in visible color was documented under ambient light to assess the system's potential as a naked-eye indicator for metabolic activity. The neutral state (pH 7.0) served as the baseline for comparison against the target acidic (pH 2.0) and alkaline (pH 12.0) environments. These transitions validate how the macroscopic appearance of the P,N-doped framework shifts in response to the metabolic acidification of the surrounding medium.

• Fluorescence sensitivity (molecular transduction): as a probe the molecular mechanisms of the sensor, the pH-dependent quenching or enhancement of photoluminescence was recorded. These optical transitions were utilized to indicate changes in the protonation state of the N- and P-rich functional groups. By monitoring these shifts at the designated pH milestones, the study will correlate the microscopic electronic changes of the nanostructures with the metabolic signatures of the bacterial environment.

### 2.4. Preparation of low degree of substitution (DS) carboxymethyl cellulose (CMC)

Cellulose was extracted from SCB as previously described.^30^ In a typical procedure, 15 g of cellulose was suspended in 400 mL of isopropyl alcohol. A 25% NaOH solution was added dropwise over 30 min, and the mixture was alkalized under stirring for 1 h at room temperature. Subsequently, 18 g of monochloroacetic acid (MCA) dissolved in isopropyl alcohol was added dropwise over 30 min. The carboxymethylation reaction proceeded under continuous stirring at 55 °C for 3.5 h. The liquid was drained, and the resulting fibrous CMC was washed in 70% methanol, filtered, and dried at 60 °C.^27,30^

The degree of substitution of the synthesized CMC was determined *via* back-titration. Initially, 2 g of CMC were dispersed in 37.5 mL of ethanol, followed by the addition of 2.5 mL of nitric acid (HNO_3_). The mixture was brought to a boil for 2 min and then stirred for 15 min at room temperature to ensure complete conversion to the acid form (H-CMC). The product was purified by washing six times with 80% ethanol (at 60 °C) to remove residual acid and then dried at 105 °C for 3 h. For the titration, 1 g of the dried H-CMC was dissolved in 100 mL of deionized water and reacted with 25 mL of 0.3 N sodium hydroxide (NaOH). The solution was heated to a boil for 15 min to facilitate the reaction. Finally, the excess NaOH was back-titrated with 0.3 N hydrochloric acid (HCl) using phenolphthalein as a visual indicator. The DS was calculated based on the milliequivalents of acid consumed, according to the standard formula:1
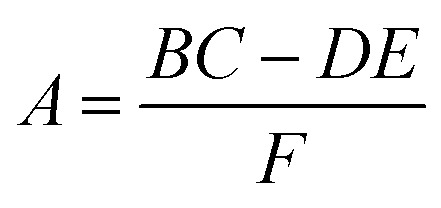
2
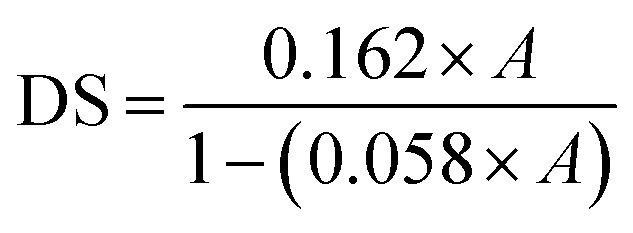
where *B*, *C* are mL and normality of NaOH; *D*, *E* are mL and normality of HCl; *F* is number of gram of CMC used; 162 = gram molecular weight of anhydroglucose unit of cellulose; 58 = net increase in molecular weight of anhydroglucose unit for each CMC substituted; and *A* is the milliequivalents of consumed acid per gram of specimen.^34^ The DS was 0.4 *via* potentiometric titration, specifically tailored to balance aqueous dispersibility.

### 2.5. Fabrication of bioactive Pickering nanoemulsions (REO@NCQDs and REO@PNCNFs)

• Formulation ratios: the aqueous phase was prepared by dispersing 1.0 g of CMC (DS = 0.4) in 30 mL of deionized water. This specific degree of substitution was selected to maintain a balance between hydrophilicity and interfacial affinity. The mixture was combined with 10 mL of the synthesized PNCNF or NCQD dispersion, creating a pre-complexed bio-ink. To this, 2 mL of Rosemary Essential Oil (REO) was added dropwise.

During the 30-minute stirring process, the partially soluble CMC chains (DS 0.4) and the P,N-doped nanostructures co-migrated to the REO/water interface. Unlike high-DS CMC, which remains primarily in the aqueous phase, the DS 0.4 variant functions as a polymeric surfactant. It interacts with the hierarchical nanoflowers to form a robust, cross-linked network that effectively stabilizes the oil droplets. The use of DS 0.4 CMC provides a unique pH-switchable barrier. Because the polymer is at the limit of its solubility, the localized production of acidic metabolites by pathogens (*e.g.*, *E. coli*) induces a rapid protonation of the remaining carboxylate groups. This causes a micro-contraction of the CMC network bringing the P,N-doped carbon sensors into closer contact with the bacterial cell walls and significantly enhancing the dual-mode optical response.

### 2.6. Structural characterization and HLB calculation

#### 2.6.1. FTIR spectroscopy and DS quantification for CMC

The chemical functionality and coordination of the synthesized samples were analyzed *via* Fourier-transform infrared (FTIR) spectroscopy using a Mattson-5000 spectrometer (Unicam, Somerset, UK). Samples were prepared using the KBr pellet technique, and spectra were recorded over the wavenumber range of 4000–400 cm^−1^ at a high resolution. To complement the titrimetric method, the Degree of Substitution (DS) was also estimated through the relative intensity of the characteristic infrared absorption bands. The calculation utilized the ratio of the absorbance of the carbonyl group to the cellulose backbone, as shown in the following equation:3
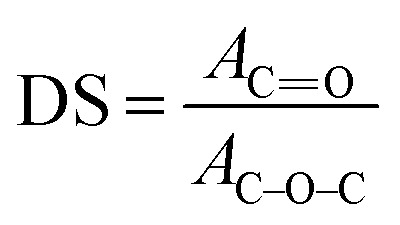
where *A*_C=O_ represents the absorbance intensity of the carboxylate group and *A*_C–O–C_ represents the absorbance of the ether linkage in the glucose rings.^35^

#### 2.6.2. Hydrophilic–lipophilic balance (HLB) for REO@NCQDs and REO@PNCNFs

The amphiphilic nature of the SCB-derived CMC, which governs its efficiency as a Pickering stabilizer, was quantified by determining its Hydrophilic–Lipophilic Balance (HLB). The HLB value was calculated according to the equation:4
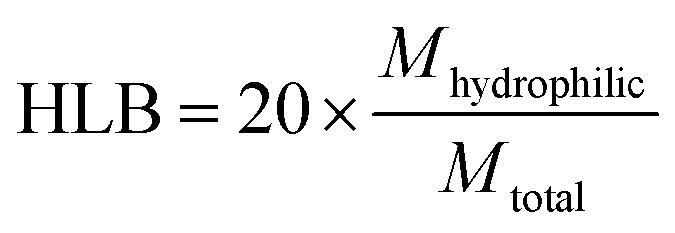
where *M*_hydrophilic_ corresponds to the molecular weight of the hydrophilic moieties and *M*_total_ represents the total molecular weight of the functionalized chains. This value is instrumental in predicting the affinity of the NCQDs or PNCNFs at the rosemary essential oil/water interface.^35,36^

### 2.7. Metabolic bacterial detection

The biological sensing potential of the REO@NCQDs and REO@PNCNFs was assessed against two model pathogens representing primary threats in clinical health and food safety: Gram-negative *Escherichia coli* and Gram-positive *Staphylococcus aureus*. The detection and inhibitory mechanisms are fundamentally linked to the interaction between the functionalized, P,N-doped surfaces and the distinct cell wall architectures of these microbes.

The bacteria were cultured at 37 °C until reaching the logarithmic growth phase. To monitor the metabolic pH signatures, a specific concentration of REO@NCQDs and REO@PNCNFs was introduced into the bacterial suspension. To investigate the effect of physical anchoring, the nanostructures were introduced to bacterial suspensions (*E. coli* and *S. aureus*) at a stabilized neutral pH. The fluorescence was monitored immediately upon contact. The resulting quenching or enhancement of the signal was attributed to the localized electronic perturbation of the P,N-doped surface groups as they adhered to the bacterial cell walls, independent of any metabolic pH shift.

### 2.8. Bacterial sensing and antibacterial efficacy assay

Bacterial suspensions of *E. coli* and *S. aureus* were prepared in nutrient broth and adjusted to an initial concentration of 1.5 × 10^8^ CFU mL^−1^ (0.5 McFarland standard). For real-time sensing, the optical response was recorded immediately upon contact (1–5 minutes). The antibacterial efficacy was evaluated *via* an endpoint CFU count after 24 hours of incubation at 37 °C.5
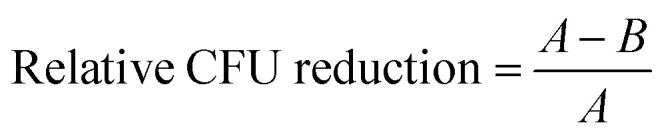
*A*: the CFU of pathogenic strain only in the control flask without any treatment. *B*: the CFU of pathogenic strain in tested flasks after applying the treating sample.

### 2.9. DFT calculations

The DFT calculations were performed using the Gaussian 09W software package at the B3LYP/6-311+G(d,p) level of theory. The structural models for NCQDs and PNCNFs were optimized using representative carbon clusters reflecting the N/P-doped domains, with implicit solvent effects (PCM model for water) included to simulate the experimental aqueous environment. These include the energy of the highest occupied molecular orbital (*E*_HOMO_), the energy of the lowest unoccupied molecular orbital (*E*_LUMO_), and the resulting energy gap (*E*_g_). The global reactivity of the system was further quantified by determining several electronic properties, including the dipole moment (*µ*), absolute hardness (*η*), absolute softness (*σ*), and chemical softness (*S*), utilizing the following equations:^37,38^

6 *E*_gap_ = (*E*_LUMO_ − *E*_HOMO_)

7
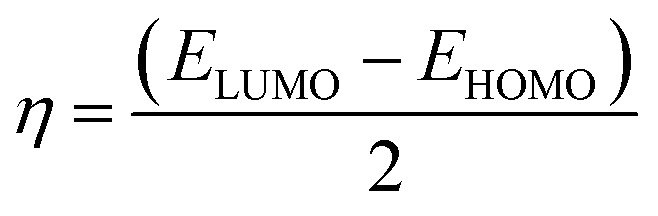
8
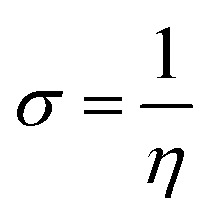
9
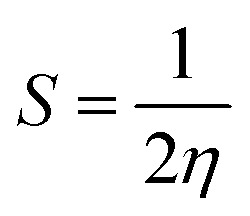


### 2.10. Fluorescent spectroscopy

Fluorescence measurements were performed using a Jasco FP-6500 Spectrofluorometer (Tokyo, Japan) equipped with a 150 W xenon arc lamp.

### 2.11. Creaming index

The physical stability of the Pickering emulsions was evaluated over a 30-day storage period at room temperature. To quantitatively characterize the stability of the formulations, the Creaming Index (CI) was determined by measuring the height of the cream layer (*H*_C_) relative to the total height of the emulsion (*H*_E_) in the storage tubes. The CI was calculated according to the following equation:10
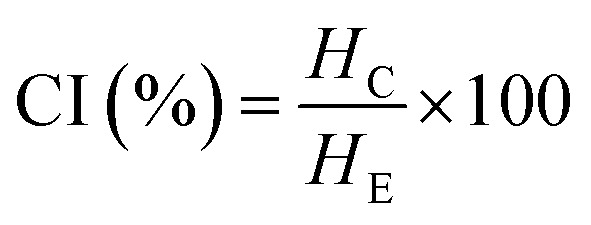
where: *H*_C_ represents the height of the separated cream layer (mm); *H*_E_ represents the total height of the emulsion (mm).

## 3. Results and discussion

### 3.1. FTIR of the prepared cellulose and carboxymethyl cellulose

**Fig. 1:**
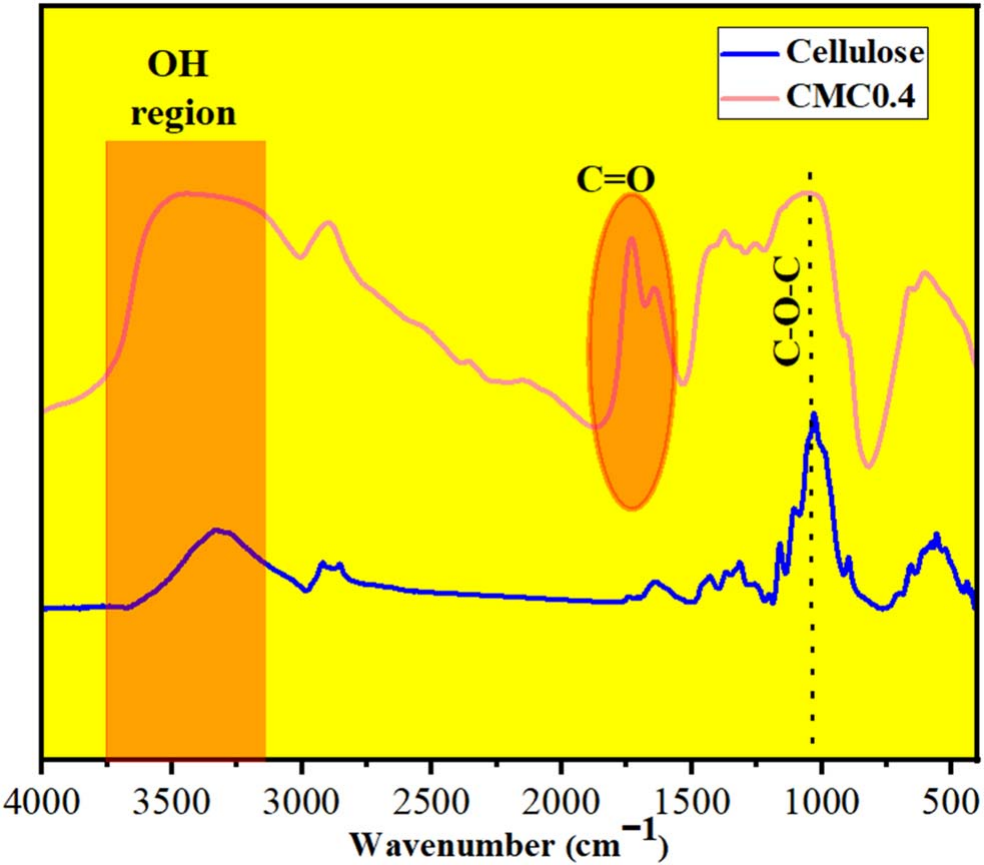
FTIR of cellulose and CMC0.4.

Cellulose showed characteristic bands at 3462, 2882, 1650, 1375, and 1040 cm^−1^ which corresponding to O–H, C–H, OH bending of the adsorbed water, CH_2_ bending and C–O–C of pyranose ring vibration, and β-glycosidic linkage between glucose units in cellulose (Fig. 1).^10^ The CMC0.4 showed the same peaks with additional peaks at 1733 and 1643 cm^−1^ corresponding to asymmetric and symmetric vibration of COO^−^ group.^30^ The presence of these peaks confirms that the chloroacetic acid treatment successfully grafted carboxymethyl groups onto the cellulose backbone. Specifically, the peak at 1733 cm^−1^ is vital; it signifies the chemical handle that allows the polymer to respond to pH shifts. In neutral conditions, these groups stay deprotonated, but as bacteria produce acid, they protonate, triggering the contraction discussed earlier.

The alignment between the potentiometric titration and the FTIR absorbance ratio method 
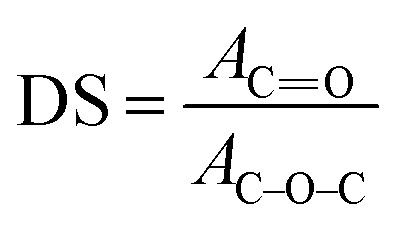
 to confirm a DS of 0.4 is a significant methodological strength. Reaching the same value *via* two independent analytical techniques (chemical and spectroscopic) provides high confidence in the material's composition. A degree of substitution (DS) of 0.4 serves as the optimal value for the stabilization of Pickering emulsions. While CMC with a higher DS (>0.7) is fully water-soluble and tends to partition into the bulk aqueous phase, the DS 0.4 variant exhibits the necessary amphiphilic balance. This degree of substitution provides sufficient hydrophobicity to facilitate migration to the rosemary oil/water, while retaining enough hydrophilicity to maintain a stable colloidal dispersion.

### 3.2. Morphological, optical properties and pH-responsive fluorescence dynamics of NCQDs and PNCNFs with DFT calculations

#### 3.2.1. Morphological observation

**Fig. 2:**
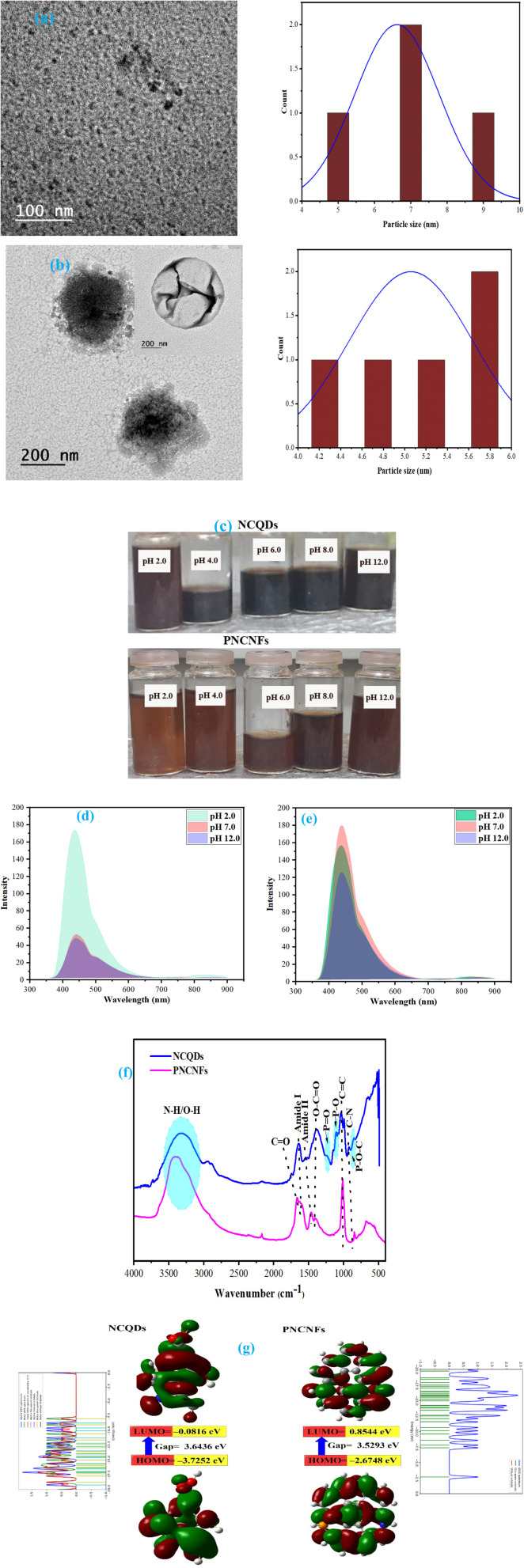
TEM analysis with particle size distribution for (a) NCQDs and (b) PNCNFs; (c) color change of NCQDs and PNCNFs under pH 2.0–12.0; and fluorescence spectroscopy for (d) NCQDs and (e) PNCNFs at pH 2.0, 7.0, and 12.0 values; (f) FTIR of NCQDs and PNCQDs; and (g) DFT calculations with DOS for NCQDs and PNCQDs.

In the architectural design of these pomegranate-derived nanomaterials, the shift from N-doped dots (NCQDs) to PNCNFs represents a sophisticated evolution in morphology control. The NCQDs are characterized as quasi-spherical, zero-dimensional (0D) nanoparticles that serve as the fundamental building blocks of the system. These dots exhibit an ultrafine and uniform size distribution, typically measuring between 5.39 and 8.14 nm. This isotropic growth is facilitated by N doping alone, which integrates into the carbon lattice without inducing the significant structural strain required for higher-dimensional assembly. In contrast, the PNCNFs hybrids demonstrate a leap into structural complexity through dual-element doping. The introduction of P, which possesses a larger atomic radius than carbon, induces localized lattice strain that disrupts isotropic growth, promoting the assembly of hierarchical nanoflowers and elongated nanofibers. This structural transition is evidenced by the evolution from quasi-spherical nanoparticles into multifaceted 3D architectures, with primary diameters ranging from 241.72 to 300.09 nm. Within these larger hybrids, a secondary population of ultra-small dots (4.14–5.61 nm) remains embedded, creating a multi-scale system that allows the material to reach fully developed hierarchical assemblies (Fig. 2a and b).

#### 3.2.2. pH-Responsiveness switchable

The pH-responsive nature of the synthesized nanostructures is visually confirmed by the distinct colorimetric transitions observed across the pH range of 2.0–12.0 (Fig. 2c). For the NCQDs, the dispersions exhibit a marked color shift, transitioning from a lighter suspension at highly acidic conditions (pH 2.0) to a darker, more concentrated coloration in neutral and alkaline environments (pH 6.0–12.0). This suggests a pH-triggered aggregation or a change in the surface charge density of the NCQDs, which alters their light absorption characteristics. In contrast, the PNCNFs demonstrate a more nuanced and dynamic colorimetric profile. At acidic conditions (pH 2.0–4.0), the PNCNF hybrids maintain a stable, translucent brown dispersion. As the pH shifts toward neutral and alkaline levels (pH 6.0–12.0), the system exhibits a noticeable darkening, indicating the protonation-induced reconfiguration of the P,N-doped polymeric framework. This visual evidence correlates directly with our DFT findings: the high global softness and polarizability of the PNCNFs allow for these rapid electronic and structural adjustments. These colorimetric shifts provide a practical, macroscopic indicator of the material's microscopic sensitivity, reinforcing our platform's capability to act as a responsive transducer for fluctuating metabolic environments where localized pH drops are the primary signal-generating event.

The selection of pH 2.0, 7.0, and 12.0 for detailed fluorescence analysis was strategically informed by our initial colorimetric characterization (Fig. 2d and e). At these specific pH levels, the NCQDs and PNCNFs exhibited the most distinct and reproducible color transitions, serving as macro-scale indicators of the materials' pH-induced electronic reconfiguration. By aligning our high-sensitivity fluorescence monitoring with these visually validated points, we ensure that our optical detection platform captures the most robust signal-generating states of the nanostructures. This integrated approach correlating macroscopic color changes with microscopic fluorometric shifts confirms that the PNCNF hybrids undergo significant charge-density adjustments at these thresholds, which facilitates the turn-on sensing response to bacterial metabolic acidification. In the characterization of these pomegranate-derived nanostructures, the optical properties reveal a profound enhancement in light-emission efficiency and a sophisticated pH-dependent response following the structural transition from dots to hierarchical hybrids. At the physiological baseline (pH 7), the NCQDs exhibit characteristic fluorescence emission peaks at 435 nm and 504 nm, representing the intrinsic electronic transitions of the quasi-spherical N-doped carbon core. However, the transition to the PNCNF hybrids at the same pH results in a significant increase in fluorescence intensity, alongside subtle spectral shifts to 439 nm and 497 nm. This substantial boost in intensity is a direct consequence of the P,N-doping and the hierarchical architecture, which facilitates enhanced radiative recombination. The introduction of P modifies the electronic density of the carbon lattice, introducing defect states that facilitate more efficient radiative recombination of electron–hole pairs. Concurrently, the hierarchical nanoflower architecture provides a significantly higher density of accessible surface functional groups compared to the quasi-spherical NCQDs (Fig. 2d and e). The dual-mode sensing capability of these materials is further elucidated by their distinct fluorescence behaviors across a wide pH range (2.0 and 12.0). For the NCQDs, the emission peaks shift to 436 nm/499 nm at pH 2.0 and 439 nm/494 nm at pH 12.0. Notably, these N-doped dots follow an intensity gradient of pH 2.0 > pH 7.0 > pH 12.0, suggesting that the high intensity in acidic conditions results from the protonation of surface functional groups which reduces non-radiative traps. In contrast, the PNCNF hybrids demonstrate a shifted intensity trend of pH 7.0 > pH 2.0 > pH 12.0, with emission peaks appearing at 438 nm/500 nm for pH 2.0 and 439 nm/500 nm for pH 12.0. This optimization at neutral pH suggests that the hierarchical petals and P-induced defect states are most electronically active at pH 7.0, whereas the slight suppression at pH 2.0 compared to pH 7.0 indicates a unique electronic reconfiguration unique to the P,N-doped framework. The spectral shifts observed across the pH range provide deep insight into the structural–optical relationship of the hybrids. The consistent red-shifts toward 439 nm suggest an increase in the size of the conjugated π-system within the larger nanoflowers, while the reorganization of oxygen-rich surface states, influenced by P atoms and pomegranate organic acids, drives the shifts in the secondary peaks toward 497–500 nm. This high-intensity, pH-variable fluorescence is a critical advantage for the sensing platform. Because the PNCNFs maintain a superior signal baseline at pH 7.0 but respond distinctly to acidic shifts, they are ideally suited to monitor metabolic pH signatures. The brighter signal of the PNCNFs ensures that the transition from neutral to acidic environments a hallmark of bacterial proliferation will be detected with much higher sensitivity and a better signal-to-noise ratio than is possible with the simpler NCQDs.

#### 3.2.3. FTIR spectroscopy of NCQDs and PNCNFs with DFT calculations

The FTIR spectroscopy in Fig. 2f showed peaks for the NCQDs and PNCNFs between 3316–3413 cm^−1^ (N–H/O–H), 1663–11749 cm^−1^ (C=O), 1603–1646 cm^−1^ (amide I), 1465–1542 cm^−1^ (amide II), 1392–1410 cm^−1^ (O–C=O), 1039–1113 cm^−1^ (C=C), 845–927 cm^−1^ (C–N), and multiple bands in the region 662–400 cm^−1^ represented the typical in-plane CH bending vibrations of aromatic compounds.^29,30^ A new peaks in NPCNFs appeared at 1240 cm^−1^ (P=O), 1101 cm^−1^ (P–O), and 858 cm^−1^ (P–O–C) which confirm the doping of P on CQDs.^39^ The relative absorbance for C–N band 
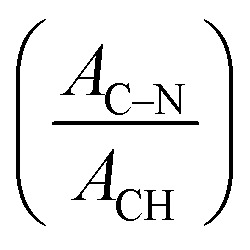
 and P–O band 
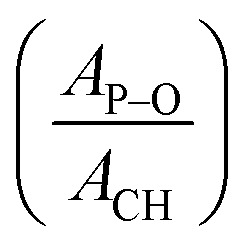
 was used to quantify the quantity of N and P, respectively. The doping efficiency for NCQDs was 1.10 for N. In addition, the doping efficiency for PNCNFs was 0.88 for N and 0.76 for P. The reduction in N's doping efficiency from 1.10 to 0.88 in the presence of P (doping efficiency 0.76) is not a loss of functionality, but rather a strategic re-wiring of the carbon surface. While the NCQDs rely on a high density of nitrogenous groups for simple surface passivation, the PNCNF hybrids utilize the synergy of both elements to create a more responsive electronic environment. The stronger H-bonding network (evidenced by the shift to 3316 cm^−1^) suggests that the surface functional groups are more tightly coupled. This is critical for the dual-mode detection: when the pH drops due to bacterial metabolism, the protonation of these tightly coupled P and N groups causes a more synchronized electronic reconfiguration than would occur in the isolated, quasi-spherical NCQDs.

The density functional theory (DFT) analysis of the NCQDs and PNCNFs reveals significant differences in their electronic architectures and potential chemical reactivities (Fig. 2g and Table 1). The frontier molecular orbital energies serve as a primary indicator of this divergence; the *E*_HOMO_ of PNCNFs (−2.6748 eV) is considerably higher than that of the NCQDs (−3.7252 eV), suggesting that the co-doped nanofibers possess a greater propensity for electron donation. This high-energy HOMO is directly linked to our experimental antibacterial performance; the enhanced electron-donating capacity facilitates the interaction with bacterial membranes, thereby disrupting cellular metabolic processes more effectively than the harder NCQD system. Furthermore, the global softness (*σ* = 0.5666 eV) of the PNCNFs provides a theoretical basis for their superior sensing performance. In the HSAB principle, high softness correlates with increased polarizability. This electronic flexibility allows the PNCNF petals to undergo rapid conformational and charge-density adjustments upon contact with acidic bacterial metabolites (lactic/acetic acid). Despite these shifts in absolute energy levels, PNCQDs maintain a remarkably narrow energy gap (*E*_g_) of approximately 3.5293 eV compared to NCQDs, which typically signifies high kinetic reactivity and efficient charge transfer capabilities. The absolute hardness (*η*) for NCQDs is calculated at 1.8218 eV, which is more than double the 1.7646 eV recorded for PNCNFs. This classifies the NCQDs as the harder system of the two, implying a more rigid electron cloud and higher chemical stability. Consequently, the increased dipole moment (2.92 debye) of the PNCNFs compared to the NCQDs (1.76 debye) confirms the high degree of electronic asymmetry that drives the observed fluorometric turn-on response. In essence, the high kinetic reactivity predicted by our DFT model is the fundamental electronic prerequisite for the interactively aggressive sensing and antibacterial behavior observed in our Pickering nanoemulsion experiments. Thermodynamic and structural characteristics are also highlighted by the ground state energies (*E*_T_) and dipole moments (*µ*). The total energy of the PNCNFs (−1228.07 a.u.) is significantly more negative than that of the NCQDs (−581.27 a.u.), reflecting the increased molecular complexity and larger atomic framework inherent in the nanofiber structure. Additionally, the dipole moment of the PNCNFs (2.92 debye) is substantially larger than that of the NCQDs (1.76 debye), suggesting that the dual-doping of P and N creates a more pronounced electronic asymmetry. This increased polarity likely enhances the interaction with polar solvents or analytes, potentially making them more effective in sensing environments. Finally, the electrophilicity index (*ω*) provides insight into the energy lowering associated with a maximal electron flow from a donor to an acceptor. The NCQDs possess a higher *ω* (0.9943 eV) compared to the PNCNFs (0.2347 eV), indicating that the quantum dots are stronger electrophiles and have a higher affinity for accepting electrons from their surroundings. This contrast suggests a functional specialization where NCQDs might excel in roles requiring electron acceptance, while the softer, more polar PNCNFs are better suited for donating charge or participating in complex surface-mediated chemical reactions.

**Table 1:** The quantum chemical NCQDs and PNCNFs

DFT	NCQDs	PNCNFs
*E*_LUMO_ (eV)	−0.0816	0.8544
*E*_HOMO_ (eV)	−3.7252	−2.6748
*E*_g_ (eV)	3.6436	3.5293
*E*_T_ (a.u.)	−581.27	−1228.07
*µ* (debye)	1.76	2.92
*σ* (eV)	0.5489	0.5666
*ω* (eV)	0.9943	0.2347
*S* (eV)	0.2744	0.2833
*η* (eV)	1.8218	1.7646

To substantiate the influence of phosphorus co-doping on the optical performance, we performed DFT-based electronic structure analysis. The comparison between the DOS spectra of the NCQDs and the PNCNFs reveals distinct electronic modifications. While the NCQDs exhibit a stable, wider-gap electronic distribution, the PNCNFs (Fig. 2g) demonstrate the emergence of a unique, localized state near −2.5 eV. This feature acts as a significant mid-gap defect state, effectively narrowing the HOMO–LUMO energy gap and facilitating more efficient charge-transfer transitions compared to the NCQD baseline. Furthermore, the high density of states in the PNCNF profile—particularly the intensified peaks between −12 eV and −16 eV confirms that phosphorus co-doping induces a more polarizable and chemically reactive electronic environment. These mid-gap states, clearly visible in the PNCNF spectrum, provide the theoretical basis for the enhanced pH-responsive turn-on fluorescence, as they offer accessible pathways for proton-induced electronic reconfiguration that are absent in the nitrogen-only doped system.

### 3.3. Morphological and dual-mode optical sensing and real-time detection of bacterial metabolic signatures for EO@NCQDs and REO@PNCNFs

#### 3.3.1. Morphological observation

**Fig. 3:**
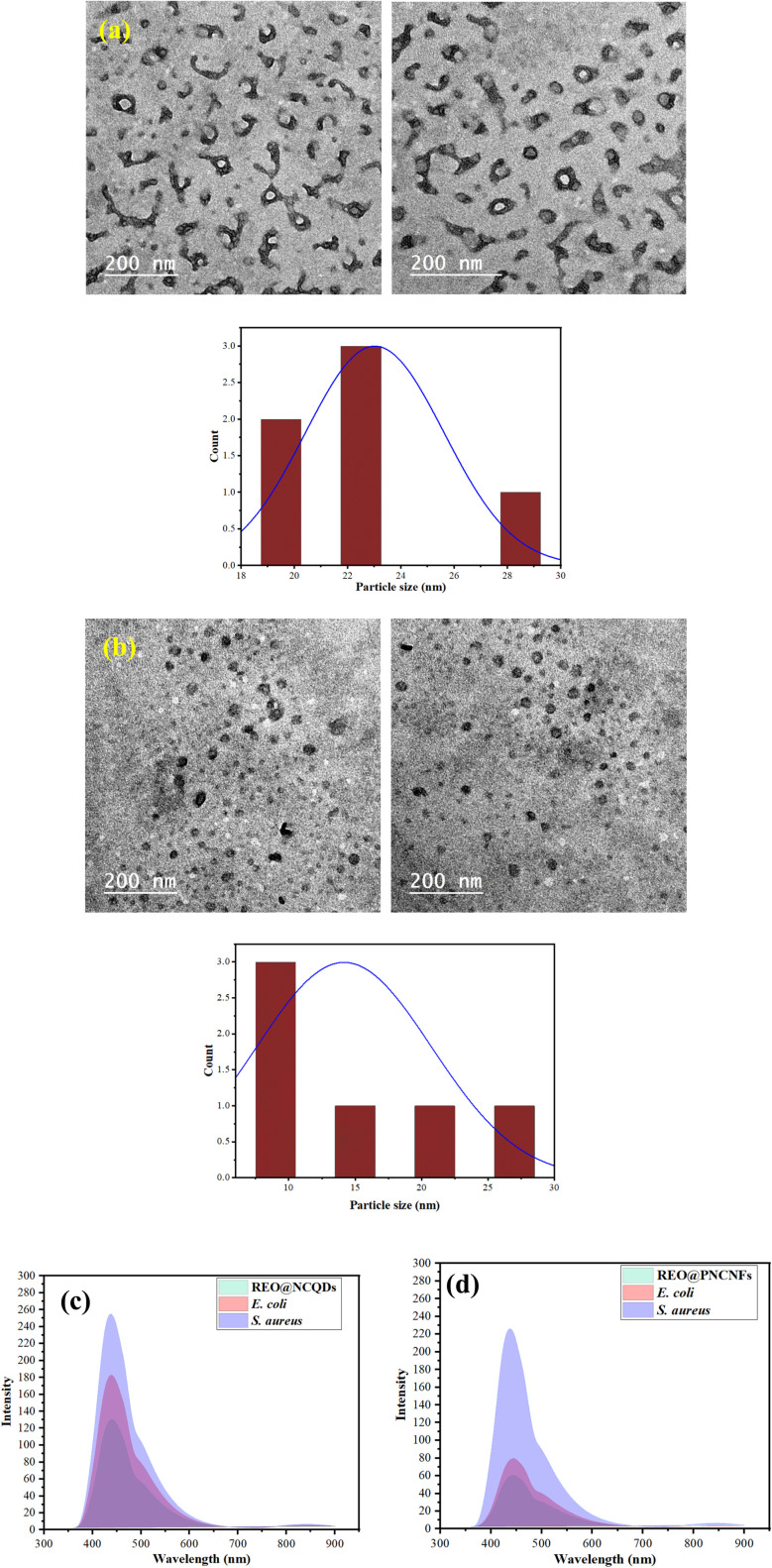
TEM analysis with particle size distribution for (a) REO@NCQDs, (b) REO@PNCNFs; and fluorescence spectroscopy for (c) REO@NCQDs and (d) REO@PNCNFs before and after contact with bacteria.

The structural development of bioactive Pickering nanoemulsions, the morphological integrity of the nanostabilizers, combined with the anionic SCB-derived CMC matrix, defines the architecture of the rosemary essential oil (REO) phase. Upon spontaneous migration to the oil–water interface, TEM analysis reveals two distinct stabilization patterns (Fig. 3a and b). In the REO@NCQD system, the quasi-spherical N-doped dots maintain their 0D structure, forming satellite clusters (19.68–27.06 nm) at the oil–water. In contrast, the REO@PNCNF system demonstrates a hierarchical arrangement, where the branched nanoflowers (8.36–24.95 nm) are embedded within the polymer matrix, providing enhanced structural stability at the oil–water. The multifaceted petals of the P,N-doped nanoflowers (8.36–24.95 nm) anchor into the REO surface, while the low-DS CMC (DS 0.4) polymer chains intertwine with the hierarchical branches. This synergy, facilitated by the amphiphilic nature of the SCB-cellulose, creates a robust mechanical barrier that prevents coalescence without the structural damage associated with high-energy sonication.

The real-time nature of this platform is evidenced by the rapid optical signal transduction observed within the first 5 minutes of contact with the bacterial inoculum (1.5 × 10^8^ CFU mL^−1^), allowing for immediate differentiation between the metabolic signatures of *E. coli* and *S. aureus*. The transition from chemical pH sensing to real-time bacterial detection is evidenced by the dramatic fluorescence response upon exposure to *E. coli* and *S. aureus*. As established in the standalone pH study, the PNCNFs are highly sensitive to acidic environments. When integrated into the emulsion, the DS 0.4 CMC acts as a metabolic concentrator. Because this specific CMC is at the limit of its solubility, it is exceptionally responsive to the localized production of lactic and acetic acid during bacterial proliferation. The resulting protonation of the CMC's carboxylate groups to (–COOH) induces a micro-contraction of the polymeric network at the oil–water.^19^ This physical shift squeezes the fluorescent PNCNF sensors into closer proximity with the bacterial cell walls, amplifying the optical signal (Fig. 3c and d).

#### 3.3.2. Fluorescence dynamics and pathogen interaction

Building upon the fundamental pH-sensitivity of the nanostructures, we integrated these materials into Pickering nanoemulsions for bacterial detection. While the pure nanostructures demonstrate a broad responsive range (2.0–12.0), the emulsion-based sensing platform is optimized for the detection of bacterial metabolic signatures, specifically capturing the localized acidification that occurs within the physiologically relevant range (pH 5.0–7.0). When these carbon nanostructures are integrated into the Pickering emulsion, their optical behavior is governed by their orientation within the CMC-stabilized interface. Interestingly, before bacterial contact, the REO@NCQDs exhibit a higher baseline fluorescence compared to the REO@PNCNF hybrids. This is likely due to the ultra-small, quasi-spherical dots being more evenly dispersed across the oil droplets, whereas the larger, hierarchical nanoflowers of the PNCNFs may experience a slight self-quenching effect due to their dense, interlocked cage structure at the interface. However, the introduction of pathogens triggers a dramatic optical reversal that highlights the superior diagnostic sensitivity of the PNCNF hybrids. Upon exposure to *Staphylococcus aureus*, however, a dramatic optical reversal occurs, demonstrating the superior diagnostic sensitivity of the PNCNF hybrids. Both systems exhibit an increase in intensity and a minor red-shift (REO@NCQDs to 439/495 nm; REO@PNCNFs to 445/500 nm). This turn-on response is driven by two synergistic mechanisms: first, the physical anchoring of the nanostructures onto the bacterial cell wall effectively passivates surface defect states, reducing non-radiative pathways and promoting radiative recombination. Second, and most importantly, the metabolic transduction of the bacterial environment. As established by our control experiments (Section 2.7), the primary driver of this signal enhancement is the metabolic acidification of the microenvironment. The localized drop in pH triggers the protonation of the carboxylate groups in the DS 0.4 CMC matrix. This induces a micro-contraction of the interfacial polymer network, physically densifying the PNCNF hybrids at the oil–water interface.

The most profound response occurs upon contact with *Escherichia coli*, where both systems reach their maximum fluorescence intensity. For the PNCNFs, this is accompanied by a strategic blue-shift to 440 nm. This heightened sensitivity to *E. coli* over *S. aureus* can be attributed to the Gram-negative bacterium's more complex metabolic output. As *E. coli* proliferates, it releases a higher concentration of acidic metabolites, which interacts uniquely with the DS 0.4 CMC. The localized drop in pH causes the partially soluble CMC network to contract, physically “pushing” the PNCNF nanoflowers into a tighter, more electronically active conformation against the bacterial membrane. The hierarchical petals of the PNCNFs provide a much larger surface area for this metabolic interaction compared to the simple NCQDs. This demonstrates that while the NCQDs are brighter in a passive state, the REO@PNCNF system is a more interactively aggressive sensor, capable of distinguishing between microbial species based on the intensity and spectral shifts of their metabolic signatures.

### 3.4. Antimicrobial efficacy and mechanistic insight (CFU analysis)

**Fig. 4:**
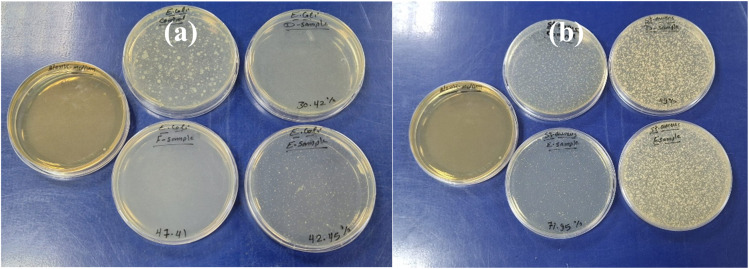
Antibacterial activity against (a) *E. coli* and (b) *S. aureus*. The control emulsion stabilized by CMC and REO alone (sample D); the REO@NCQDs (sample E); and the REO@PNCNF (sample F) system.

The quantitative evaluation of antimicrobial performance using the CFU method reveals a compelling relationship between the physical shape of the nanostabilizers and their bactericidal efficiency. As shown in Fig. 4, the journey from a basic emulsion to a hierarchical nanophotonic system significantly alters the survival rates of both *E. coli* and *S. aureus*. The study began with the control emulsion (sample D), composed of rosemary essential oil (REO) stabilized only by the SCB-derived CMC matrix. This baseline formulation presented a polarized response: while it was surprisingly effective against the Gram-negative *E. coli* (47.41% inhibition), it was largely ineffective against the Gram-positive *S. aureus*, managing only an 11.18% reduction. This disparity highlights a classic challenge in essential oil delivery; without a specialized carrier, the oil can penetrate the thin lipid membrane of *E. coli* but is physically hindered by the thick, mesh-like peptidoglycan layer that characterizes *S. aureus*. The introduction of quasi-spherical N-doped dots in sample E (REO@NCQDs) shifted this dynamic toward a more balanced, yet still limited, inhibitory profile. Against *S. aureus*, the efficacy jumped to 49.00%, proving that even zero-dimensional dots help anchor the oil droplets to the bacterial surface. Interestingly, the inhibition of *E. coli* dropped to 30.42%. We hypothesize that the dense packing of these uniform dots at the droplet boundary may create a diffusion-limiting barrier, illustrating a trade-off between surface adhesion and rapid oil release. The breakthrough occurs with sample F (REO@PNCNF), where the transition to 3D hierarchical nanoflowers pushes the system to its maximum performance, achieving an inhibition rate of 71.75% against *S. aureus* and 42.45% against *E. coli*. The increasing in efficacy against *S. aureus* compared to the control is particularly remarkable. We propose that the multifaceted petals of the P,N-doped nanoflowers act as high-density docking sites that facilitate localized oil delivery directly onto the pathogen's membrane. Furthermore, the electronic reconfiguration introduced by P co-doping supported by our DFT calculations of increased absolute softness 0.57 eV suggests a localized charge environment that may perturb the bacterial membrane potential. While further biological assays are required to confirm this membrane-disruptive effect, these results indicate that the hierarchical architecture of the PNCNFs is the critical element required to transform a simple essential oil emulsion into a high-tier, bioactive diagnostic platform.

### 3.5. Interfacial mechanics, stability and visual evolution

#### 3.5.1. Hydrophilic–lipophilic balance (HLB)

The transition from quasi-spherical NCQDs to hierarchical PNCNF hybrids is not only a morphological evolution but a fundamental shift in surface thermodynamics. This shift was quantified through the Hydrophilic–Lipophilic Balance (HLB), which serves as a predictor for the material's behavior at the Rosemary Essential Oil (REO)/water. The HLB value for PNCNFs was calculated at 8.95, a significant increase from the 5.57 recorded for NCQDs. This divergence is rooted in the molecular re-wiring induced by dual-element doping. While the NCQDs rely primarily on nitrogenous surface states (1.10), the PNCNFs integrate P (0.76), which contributes highly polar P–O and P–OH moieties to the *M*_hydrophilic_ component. To contextualize these values, the HLB of commercial emulsifiers such as Span 85 (1.8), Span 80 (4.3), and Span 40 (6.7) are notably lower.^35,40^ The superior HLB of the PNCNFs (8.95) places them in the optimal range for oil-in-water (O/W) emulsification, ensuring higher polar character compared to traditional lipophilic surfactants. The high HLB of the PNCNF hybrids provides a dual advantage in the context of the Pickering nanoemulsion. First, the enhanced hydrophilicity ensures that the P,N-doped framework is easily dispersible in the aqueous phase, preventing the burying of active sites within the oil droplets. Second, this polar surface architecture creates a more responsive smart interface. Because the PNCNFs are more hydrophilic and polarizable (as evidenced by the high global softness in DFT calculations), they exhibit a heightened sensitivity to the localized protonation changes triggered by bacterial metabolic pH signatures. Consequently, the PNCNF-stabilized system acts as a more interactively aggressive platform. The synergy between the DS 0.4 CMC and the high-HLB PNCNFs allows the antimicrobial compounds in the REO to remain bioavailable at the interface, while simultaneously providing a high-density field of P and N groups for the real-time fluorometric and colorimetric detection of pathogens. This thermodynamic alignment effectively bridges the gap between the organic antimicrobial reservoir and the aqueous biological environment, setting a new benchmark for multifunctional biosensing.

#### 3.5.2. Stability and visual observation

**Fig. 5:**
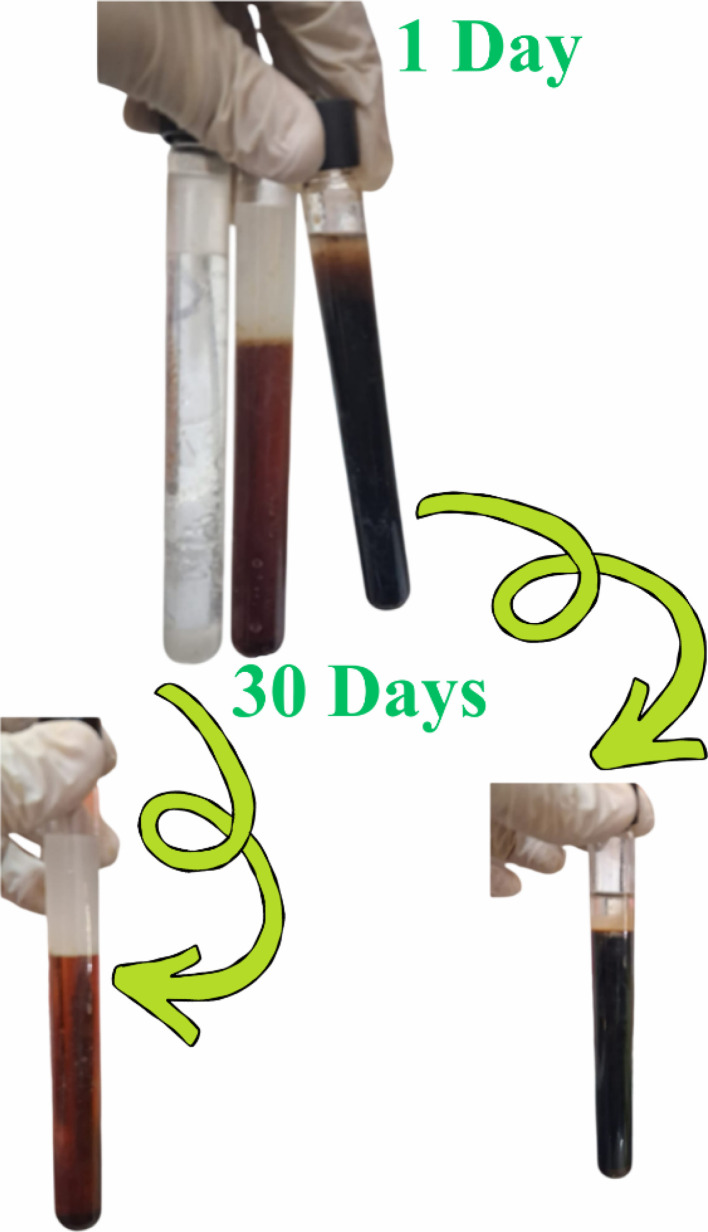
Visual observation of the REO@NCQDs and the REO@PNCNF emulsions at 1 day and after 30 days.

The ultimate validation of a Pickering stabilizer lies in its ability to prevent phase separation over extended shelf-lives. As demonstrated in Fig. 5, the visual comparison between the initial formulation (day 1) and the long-term observation (day 30) reveals an extraordinary degree of stability for both REO@NCQDs and REO@PNCNF. Both samples exhibit remarkable resistance to the typical destabilization mechanisms, such as creaming, sedimentation, or coalescence, which often plague essential oil emulsions within the first few days of storage. This month-long stability is primarily driven by the interfacial anchoring provided by the SCB-derived DS 0.4 CMC. Because this specific CMC is engineered to be at the limit of its solubility, it acts as a polymeric glue that traps the carbon nanostructures at the surface of the rosemary oil droplets. In REO@PNCNF, the hierarchical 3D nanoflowers and nanofibers create a multifaceted mechanical barrier a caging effect that physically prevents the droplets from merging. The visual consistency of the dark, nutrient-rich hues in both samples after 30 days indicates that the P,N-doped materials remain securely positioned at the interface rather than leaching into the aqueous phase. For a smart packaging application, this 30-day stability window is highly significant, as it matches or exceeds the typical shelf-life requirements for many fresh food products. It confirms that the pomegranate and sugarcane-derived platform is not just a laboratory concept, but a functional, stable technology ready for real-world antimicrobial and sensing tasks.

To substantiate these visual observations, we calculated the Creaming Index (CI) for both systems over the 30-day storage period. The results quantitatively confirm the superior stability of the hierarchical architecture: the REO@NCQDs system exhibited a CI of 31.57%, whereas the REO@PNCNF formulation demonstrated a significantly lower CI of 15.38%. This substantial reduction in the creaming rate for the REO@PNCNF emulsion provides a clear numerical mandate for the enhanced stabilization efficiency of the 3D nanoflower–nanofiber network. While the NCQDs provide a baseline for droplet entrapment, the lower CI of the PNCNF system underscores that the caging effect facilitated by the hierarchical geometry is more effective at arresting droplet movement and preventing the upward migration of the oil phase. These quantitative indicators provide robust evidence that the nanostructured stabilizers are not merely suspended but are firmly and uniformly anchored at the oil–water interface throughout the shelf-life of the emulsion.

### 3.6. Limitations and future perspectives

While the PNCNF-emulsion platform demonstrates high sensitivity and robust pH-responsive optical signaling, we acknowledge that the mechanistic decoupling of metabolic activity from passive interfacial interactions remains a complex challenge. Our current findings establish the pH-triggered fluorescence turn-on mechanism as a proof-of-concept for detecting microbial presence; however, fully characterizing the platform's selectivity requires further rigorous validation.

Future work will expand upon these results through the implementation of dedicated control studies, specifically utilizing bacteria-free culture media and heat-inactivated bacterial cells as negative control groups. These experiments are essential for three primary reasons:

• Discrimination of metabolic signals: by comparing the fluorescence response of viable bacteria against heat-inactivated cells, we can definitively isolate the contribution of metabolically induced acidification from the passive adsorption of nanostructures onto the bacterial cell wall.

• Mitigation of non-specific interference: evaluating the sensor response in sterile culture media will quantify the influence of background medium components, ensuring that the detected signal is exclusively a result of bacterial metabolic byproducts rather than interference from growth broth constituents.

• Refinement of surface interactions: further studies will examine the competitive binding at the emulsion to distinguish between the turn-on response triggered by pH fluctuations and potential non-specific interactions between the P,N-doped polymeric framework and cell surface proteins.

• Quantitative calibration: future work will establish standardized calibration curves correlating bacterial colony-forming unit (CFU) concentrations with real-time fluorescence intensity, allowing for the quantification of microbial load in addition to the current qualitative detection.

These mechanistic investigations will be instrumental in bridging the gap between current laboratory-scale sensing and the eventual translation of this technology into practical, high-fidelity diagnostic applications for real-time environmental and food quality monitoring.

## 4. Conclusions

This study successfully demonstrated a dual-waste valorization strategy, transforming pomegranate skin and sugarcane bagasse into a high-performance, smart nanophotonic platform for pathogen detection. By employing microwave-assisted hydrothermal carbonization, we engineered a sophisticated structural evolution from 0D quasi-spherical N-doped dots (NCQDs) to 3D hierarchical P and N co-doped nanoflower/fiber hybrids (PNCNFs). The integration of P was found to be the critical structural director, inducing lattice strain and electronic reconfiguration that shifted the material's hydrophilic–lipophilic balance (HLB) from 5.57 to an optimal 8.95. This elevated HLB, surpassing commercial emulsifiers like Span 40 and 80, ensured superior interfacial anchoring within the Pickering nanoemulsions. Combined with the tailored low-DS (0.4) carboxymethyl cellulose (CMC), the PNCNF hybrids formed a stable, responsive bio-ink capable of sitting precisely at the oil/water interface. The PNCNFs provided synchronized colorimetric and fluorometric signaling, allowing for both naked-eye screening and high-sensitivity molecular transduction of bacterial presence. The high surface-to-volume ratio of the nanoflower petals facilitated the real-time detection of metabolic pH signatures, specifically responding to the acidification caused by *E. coli* and *S. aureus*. The hierarchical hybrids not only detected pathogens but also enhanced the inhibitory effect of rosemary essential oil (REO) through a pH-switchable interfacial contraction. In conclusion, this research bridges the gap between agricultural waste management and advanced nanophotonics. The resulting P,N-doped carbon nanostructures offer a robust, sustainable, and interactively aggressive tool for real-time food safety monitoring and clinical diagnostics, establishing a new paradigm for multifunctional green biosensors.

## Author contributions

Hebat-Allah S. Tohamy: writing – review and editing, writing – original draft, visualization, validation, supervision, software, resources, project administration, methodology, investigation, funding acquisition, formal analysis, data curation, conceptualization.

## Conflicts of interest

The authors declare that they have no known competing financial interests or personal relationships that could have appeared to influence the work reported in this paper.

## Data Availability

All experimental data supporting the findings of this study are included within the article. Additional datasets generated and analyzed during the current study are available from the corresponding authors upon reasonable request.

## References

[cit1] Arustamov V. (2024). *et al.*, Plasma Vacuum-Arc Treatment Technology for the Metal Pipe Surfaces of Solar Thermal Power Plants. Appl. Sol. Energy.

[cit2] Bulgakova N. M. (2014). *et al.*, Impacts of ambient and ablation plasmas on short-and ultrashort-pulse laser processing of surfaces. Micromachines.

[cit3] Chanturiya V. A., Bunin I. Z. (2022). Advances in pulsed power mineral processing technologies. Minerals.

[cit4] Chattopadhyay R. , Advanced Thermally Assisted Surface Engineering Processes, Springer, 2004

[cit5] Tohamy H.-A. S. , Quantum dots as an emerging nanocarrier for drug delivery, in Quantum Dot Nanocarriers for Drug Delivery, 2025, pp. 363–384

[cit6] Tohamy H.-A. S. (2025). Artistic anti-counterfeiting with a pH-responsive fluorescent ink using DFT and molecular electrostatic potential mapping insights. Sci. Rep..

[cit7] Mohamed F. E.-Z. S., Tohamy H.-A. S., El-Sakhawy M. (2025). Hepatoprotective activity of bio-fabricated carbon quantum dots-decorated zinc oxide against carbon tetrachloride-induced liver injury in male rats. BMC Pharmacol. Toxicol..

[cit8] Tohamy H.-A. S. (2025). Correction: Speedy synthesis of magnetite/carbon dots for efficient chromium removal and reduction: a combined experimental and DFT approach. Emergent Mater..

[cit9] Tohamy H.-A. S. (2025). Fullerene-functionalized cellulosic hydrogel biosensor with bacterial turn-on fluorescence response derived from carboxymethyl cellulose for intelligent food packaging with DFT calculations and molecular docking. Gels.

[cit10] Tohamy H.-A. S. (2025). Microwaved Schiff base dialdehyde cellulose-chitosan hydrogels for sustained drug release with DFT calculations. BMC Chem..

[cit11] Tohamy H.-A. S. (2026). *et al.*, Controlled Release of 4-Aminoacetophenone by Janus Amphiphilic Graphene Oxide Initiates Antitumor Activity and Apoptotic Breast Cancer Cell Death In Vitro. BioNanoScience.

[cit12] Pirzadeh M. (2021). *et al.*, Pomegranate as a source of bioactive constituents: A review on their characterization, properties and applications. Crit. Rev. Food Sci. Nutr..

[cit13] Gosset-Erard C. (2021). *et al.*, Identification of punicalagin as the bioactive compound behind the antimicrobial activity of pomegranate (Punica granatum L.) peels. Food Chem..

[cit14] Jurenka J. (2008). Therapeutic applications of pomegranate (Punica granatum L.): a review. Alternative Med. Rev..

[cit15] Zuccari G. (2020). *et al.*, Formulation strategies to improve oral bioavailability of ellagic acid. Appl. Sci..

[cit16] Rahul P. (2025). *et al.*, Recent advances in encapsulation of pomegranate peel extract and combination of wall materials: a review of encapsulation technologies, characterization and applications in the food industry. Sustainable Food Technol..

[cit17] Motawe E. H. (2023). *et al.*, Nano-Capsulation of Ginger, Red Cabbage and Broccoli Ball Mill Extracts As Sources of Anti-Oxidant and Anti-Cancer and Application in Lentil Soup Powder. Egypt. J. Chem..

[cit18] Yadav S. , *et al.*, Biogenic synthesis of nanomaterials: bioactive compounds as reducing, and capping agents, in Biogenic Nanomaterials for Environmental Sustainability: Principles, Practices, and Opportunities, Springer, 2024, pp. 147–188

[cit19] Mondal R. (2009). *et al.*, Influence of anion on the coordination mode of a flexible neutral ligand in Zn(II) complexes: from discrete zero-dimensional to infinite 1D helical chains, 2D nanoporous bilayer networks, and 3D interpenetrated metal–organic frameworks. Cryst. Growth Des..

[cit20] Shi J. (2020). *et al.*, Smart textile-integrated microelectronic systems for wearable applications. Adv. Mater..

[cit21] Shahzadi S. (2024). *et al.*, A review on synthesis of MOF-derived carbon composites: innovations in electrochemical, environmental and electrocatalytic technologies. RSC Adv..

[cit22] Wang H.-m. (2013). *et al.*, Phosphorus-doped graphene and (8, 0) carbon nanotube: structural, electronic, magnetic properties, and chemical reactivity. Appl. Surf. Sci..

[cit23] Cruz-Silva E. (2009). *et al.*, Electronic transport and mechanical properties of phosphorus- and phosphorus–nitrogen-doped carbon nanotubes. ACS Nano.

[cit24] El-Nasharty M., El-Sakhawy M., Tohamy H.-A. S. (2025). Temperature responsive aluminum manganese doped carbon dot sensors for enhanced electrical conductivity with DFT calculations. Sci. Rep..

[cit25] Tohamy H.-A. S. (2025). A novel anthocyanins hydroxyethyl cellulose film for intelligent chicken meat packaging with mechanical study, DFT calculations and molecular Docking study. Sci. Rep..

[cit26] Tohamy H.-A. S. (2025). A novel natural chromogenic visual and luminescent sensor platform for Multi-Target analysis in strawberries and shape memory applications. Foods.

[cit27] Tohamy H.-A. S. (2025). Beet root carbon dots cellulose sulfate film as a novel naked eye pH sensor for chromium and bacterial detection in tomatoes. Sci. Rep..

[cit28] Tohamy H.-A. S. (2025). Amylopectin xerogel with onion based sulfur nitrogen doped carbon quantum dots as a chemosensor for chromium and biosensor for microbial spoilage in tomatoes. Sci. Rep..

[cit29] Tohamy H.-A. S. (2025). Novel onion derived sulfur nitrogen carbon quantum dots/cellulose sulfate naked-eye pH-sensitive sensor for chromium, bacterial and fungal detection in green beans. J. Food Meas. Char..

[cit30] Tohamy H.-A. S., El-Sakhawy M., Elwan A. M. (2026). In situ synthesis of aluminum/copper-doped carbon dots from magnetite graphene oxide-carboxymethyl cellulose-2-acrylamido-2-methyl-1-propanesulfonic acid hydrogel and their electrical characterization. RSC Adv..

[cit31] Qin W. (2020). *et al.*, Microbe-Mediated Extracellular and Intracellular Mineralization: Environmental, Industrial, and Biotechnological Applications. Adv. Mater..

[cit32] Loto C. A. (2017). Microbiological corrosion: mechanism, control and impact—a review. Int. J. Adv. Manuf. Technol..

[cit33] Tohamy H.-A. S. (2026). Aggregation-induced emission garlic peel carbon dots infused-carrageenan/whey protein emulsion for apple spoilage biosensing. J. Food Meas. Char..

[cit34] Tohamy H.-A. S. (2025). Novel intelligent naked-eye food packaging pH-sensitive and fluorescent sulfur, nitrogen-carbon dots biosensors for tomato spoilage detection including DFT and molecular docking characterization. Int. J. Biol. Macromol..

[cit35] Tohamy H.-A. S. (2025). *et al.*, Antimicrobial Plectranthus amboinicus emulsions prepared with amphiphilic cellulose stearate. Euro-Mediterr. J. Environ. Integr..

[cit36] El-Sakhawy M. (2025). *et al.*, Amphiphilic Carboxymethyl Cellulose Stearate for Pickering Emulsions and Antimicrobial Activity of Chrysanthemum Essential Oil. J. Renewable Mater..

[cit37] Tohamy H.-A. S. (2026). Aggregation-induced emission garlic peel carbon dots infused-carrageenan/whey protein emulsion for apple spoilage biosensing. J. Food Meas. Char..

[cit38] Tohamy H.-A. S. (2026). Novel hollow carbon dots from cactus cladodes Peel as a qualitative ‘turn-on’ fluorescence and naked-eye detection of the flammable cyclohexane gas sensor. Discover Mater..

[cit39] Kalaiyarasan G., Joseph J., Kumar P. (2020). Phosphorus-Doped Carbon Quantum Dots as Fluorometric Probes for Iron Detection. ACS Omega.

[cit40] El-Sakhawy M. (2025). *et al.*, Amphiphilic Carboxymethyl Cellulose Stearate for Pickering Emulsions and Antimicrobial Activity of Chrysanthemum Essential Oil. J. Renewable Mater..

